# Metasin—An Intra-Operative RT-qPCR Assay to Detect Metastatic Breast Cancer in Sentinel Lymph Nodes

**DOI:** 10.3390/ijms140712931

**Published:** 2013-06-24

**Authors:** Salma Al-Ramadhani, Priya Sai-Giridhar, Dilushana George, Preethi Gopinath, Evdokia Arkoumani, Samar Jader, Maryse Sundaresan, Roberto Salgado, Dennis Larsimont, Stephen A. Bustin, Vasi Sundaresan

**Affiliations:** 1 Cellular Pathology, Princess Alexandra Hospital NHS Trust, Harlow, Essex CM20 1QX, UK; E-Mails: afash49@hotmail.com (S.A.-R.); priya.sai-giridhar@wales.nhs.uk (P.S.-G.); dilushana.george@pah.nhs.uk (D.G.); parvapree@aol.com (P.G.); evdokia.arkoumani@pah.nhs.uk (E.A.); samar.jader@pah.nhs.uk (S.J.); 2 Breast Unit, Prince Philip Hospital Bryngwynmawr, Llanelli Carmarthenshire SA14 8QF, UK; 3 Department of Histopathology, Southend University Hospital NHS Trust, Southend, Essex SS0 0RY, UK; E-Mail: maryse.sundaresan@southend.nhs.uk; 4 Department of Pathology, Jules Bordet Institute, Brussels 1000, Belgium; E-Mails: roberto.salgado@bordet.be (R.S.); denis.larsimont@bordet.be (D.L.); 5 Postgraduate Medical Institute, Faculty of Health, Social Care and Education, Anglia Ruskin University, Chelmsford, Essex CM1 1SQ, UK; E-Mail: stephen.bustin@anglia.ac.uk

**Keywords:** Metasin, CK19, sentinel node, metastatic, GeneSearch, intra-operative, OSNA, NICE, Mammaglobin, Cepheid, qPCR, POC, breast, cancer, PBGD, axillary, immunostains, MGB

## Abstract

Nodal status is one of the most important prognostic factors in breast cancer. Established tests such as touch imprint cytology and frozen sections currently used in the intra-operative setting show variations in sensitivity and specificity. This limitation has led to the development of molecular alternatives, such as GeneSearch, a commercial intra-operative real-time quantitative Polymerase Chain Reaction (RT-qPCR) assay that allows the surgeon to carry out axillary clearance as a one-step process. Since GeneSearch has been discontinued, we have developed the replacement Metasin assay, which targets the breast epithelial cell markers CK19 and mammaglobin mRNA and identifies metastatic disease in sentinel lymph nodes. The optimised assay can be completed within 32 min (6 min for RNA preparation and 26 min instrument run time), making its use feasible in the intraoperative setting. An analysis by Metasin of 154 archived lymph node homogenates previously analysed by both parallel histology and GeneSearch showed concordance for 148 cases. The sensitivity and specificity of Metasin compared with GeneSearch were 95% (CI 83%–99%) and 97% (CI 91%–99%) respectively; compared with histology they were 95% (CI 83%–99%) and 97% (CI 91%–99%), respectively. The sensitivity and specificity of GeneSearch compared with histology were 90% (CI 77%–96%) and 97% (CI 93%–99%) respectively. The positive predictive value of Metasin was 90% and negative predictive value was 98% for both histology and GeneSearch. The positive predictive value of GeneSearch was 92% and the negative predictive value was 97% compared to histology. The discordance rates of Metasin with both GeneSearch and histology were 3.89%. In comparison, the discordance rate of GeneSearch with histology was 4.5%. Metasin’s robustness was independently evaluated on 193 samples previously analysed by GeneSearch from the Jules Bordet Institute, where Metasin yielded comparable results.

## 1. Introduction

The most important prognostic factor in breast cancer is the presence or absence of disseminated tumours in the axillary lymph nodes [1,2] and the spread of cancer to the sentinel lymph nodes, whilst not classifying the patient as having M1 disease, is indicative of a poorer prognosis. Hence the assessment of sentinel lymph node biopsies is the standard of care for staging the axilla [3–5], and its intra-operative examination offers the advantage of allowing an immediate axillary dissection of a positive node. This avoids the need for a second operation with its associated anxiety and risk to the individual patient, releases theatre time for more operations, cuts down on the demand for surgical beds and thereby, reduces costs to the National Health Service (NHS).

However, conventional intra-operative methods, such as frozen section and touch cytology, are time- and labour intensive and have a wide range of sensitivity, ranging from 59% to 88.2% for frozen sections and from 40% to 58.3% for touch imprint cytology [6–8]. This has led to the evaluation of real time quantitative PCR (qPCR)-based methods, which are sensitive, specific, rapid, relatively inexpensive and can be automated, making their use in the intra-operative setting practical. Furthermore, whereas histology only assesses a representative section of the tissue and therefore introduces sampling error, qPCR analysis can potentially be carried out on the whole node for the presence of markers of interest. The GeneSearch BLNA assay (Veridex LLC, WARREN, NJ, USA) is a now discontinued commercial intra-operative qPCR assay that detected the presence of tumour-specific markers cytokeratin-19 (CK-19) and mammaglobin (MGB) in sentinel lymph nodes [5,9–11]. If either one or both of the markers were positive, the node was considered positive and an axillary clearance would be carried out. Porphobilinogen deaminase (PBGD) was used as a positive control for successful RNA extraction. Despite its proven clinical usefulness, preliminary Federal Drug Administration (FDA) approval and use in the UK and Europe, the assay was withdrawn, mainly due to insufficient implementation in the US home market [12].

Consequently, we set out to develop a replacement assay, termed Metasin, using the same markers, but with freely available commercial reagents and capable of running on any thermocycler. Importantly, publication of primer and probe details will enable biomedical scientists to optimise the assay within their own institutions. The primers and probe sequences, PCR master mixes and run conditions were optimised and the assay was validated to generate an assay that matched the performance of the GeneSearch assay [5,9–11]. This report compares the performance of the new Metasin assay with both conventional histological methods that provide a ‘gold standard’ as well as the GeneSearch assay for the assessment of archived lymph node lysates from a cohort of 154 cases.

## 2. Results

### 2.1. Patient Demographics

Details listing the patient demographics and relevant clinical information were retrospectively collected and are presented in Table 1. No patient demographics were available for the samples from the Jules Bordet Institute (JBI).

### 2.2. Characteristics of the PCR Assay

Metasin PCR efficiencies for CK19, MGB and PBGD-specific were 97.3%, 100% and 100%, respectively (results not shown). A comparison of amplification reactions carried out as either monoplex and multiplex reactions resulted in an average difference in quantification cycle (ΔCq) of 1.5, suggesting little interference between the nine primer/probe combinations. Analysis of PCR products on agarose gels resulted in single bands of the expected size and the absence of smearing, indicating an optimised PCR reaction (Figure S1). Since pseudogenes are known to exist for CK19, its sequence was aligned with those of the pseudogenes, demonstrating the specificity of the primer and probe used (Figure S2). Absence of pseudogene amplification was also demonstrated empirically by using DNA from 100 colon tumours as templates in the Metasin PCR reaction. No amplification signals were detected in either the CK19 or MGB reactions and the fluorescence detected in the PBGD reaction was below the cut-off threshold established previously. Gel electrophoresis of the PCR reactions showed a series of smears (results not shown).

### 2.3. Cut-Off Values for Positive and Negative Nodes and Macro- and Micro-Metastases

**Figure 1:**
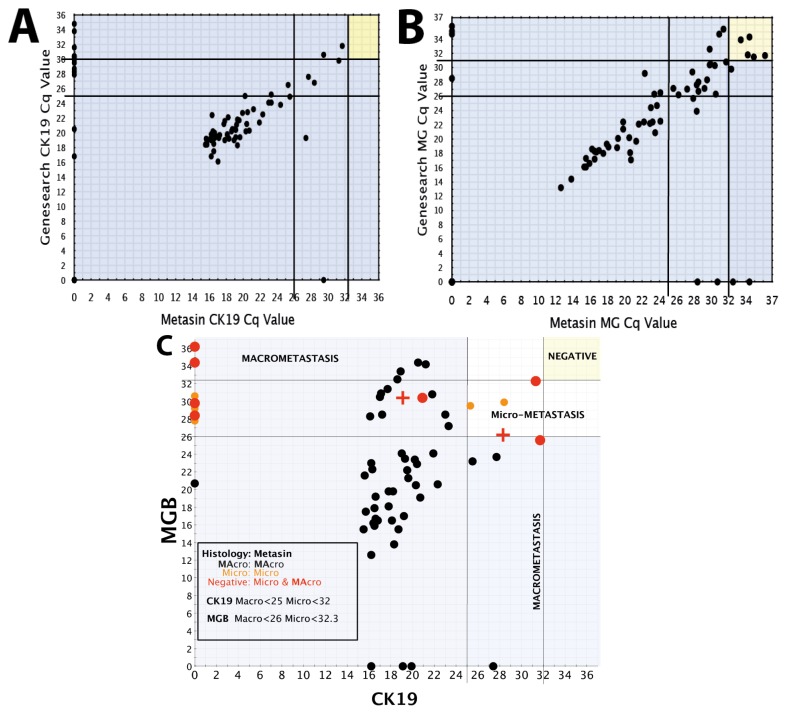
Determining the cut-off Cq values for positive and negative nodes and macro and micro metastases. (**A**) & (**B**) Scatter plots of Cq values GeneSearch data points (*x*-axis) compared to that obtained with Metasin (*y*-axis) for A: CK19 and B: MGB; (**C**) Scatter plot of Cq values obtained with Metasin Cq values for CK19 and MGB are illustrated in the x and y axis respectively. The numbers denote the Cq values obtained from the Cepheid Smart Cycler after 36 cycles by Metasin. The data points representing Cq values for nodes within the blue shaded area represent macros by Metasin, whilst data points in the clear region are micros by Metasin. Cq values in the yellow shaded area are negative or represent isolated tumour cells. The black data points represent nodes that are macros by both histology and GeneSearch (*n =* 47). The orange data points represent concordant micros by both histology and GeneSearch (*n =* 5). The red data points represent discordant histologically negative nodes that were scored as micro (*n =* 7, red circles) and macro (*n =* 2, red crosses) by GeneSearch. The inset shows the Cq cut off values for macro and micros by Metasin for CK19 and MGB.

Once all concordant positive cases were identified, histology and GeneSearch results were used to group the nodes into macrometastases and micrometastases (Figure 1). Extrapolation from the scatterplot identified cut-off quantification cycle (Cq) values for positive and negative nodes as ≤32 for CK19 and ≤32.3 for MGB. The cut-off Cq values for macrometastases were identified as ≤25 for CK19 and/or ≤26 for MGB. The cut-off Cq values for micrometastases were identified as between 25.1 and 32 for CK19 and/or between 26.1 and 32.3 for MGB. This is similar to the GeneSearch criteria, that record macrometastases as having a Cq value of <25 for CK19 and <26 for MGB, with micrometastases having Cq values between 25 and 30 for Ck19 and 26 and 31 for MGB [5,9–11]. The standard histological criteria for reporting of macrometastases are tumour deposits greater than 2 mm and for micrometastases between 0.2 and 2 mm [13–15].

### 2.4. Comparison of Metasin with Histology

A total of 350 nodes from 154 cases were tested using Metasin and compared with histology (Tables 2 and 3). 37 cases (24%) containing 61 positive nodes were positive for both Metasin and histology. Of these, 50 (81.9%) were macrometastases and eight (13%) micrometastases by both Metasin and histology (Table 4). Three nodes were positive overall but with differing tumour volumes (Table 4). 111 cases (72%) containing 280 nodes were negative for both.

There were initially 10 (6.49%) discordant cases (Table 2, upper panel). Eight were false positives, defined as histology-negative and Metasin-positive (H− M+) and two were false negatives, defined as histology-positive and Metasin-negative (H+ M−).

An additional histological assessment of the eight false positives at deeper levels and staining with MNF116, a pancytokeratin marker, revealed four to be positive showing micrometastases, reducing the number of false positive discordant cases to four (see Table 2, lower panel). When the four cases with micrometastases on deeper levels were tested with GeneSearch, three also tested positive but one was negative.

The Metasin assay was rerun on the two false negative (H+ M−) cases and both were found to be consistently negative for Metasin (Table 2). When tested with GeneSearch, one of these tested positive the other negative; further descriptions of these two cases are provided in the discussion section. The overall case-based discordant rate after discordance is 3.89 percent (Tables 2 and 3).

Consequently, the sensitivity of Metasin compared with histology is 95% (37/39, 95% CI 83%–99%) and the specificity is 97% (CI 91%–99%). The discordance rate of Metasin compared with histology is 3.89%. Metasin has a positive predictive value (PPV) of 90% and a negative predictive value (NPV) of 98%. The sensitivity of GeneSearch compared with histology is 90% (77%–96%, 95% CI) and its specificity is 97% (CI 93%–99%), PPV is 92% and NPV is 97%. The discordance rate is 4.5%.

### 2.5. Comparison of Metasin with GeneSearch

A total of 350 nodes from 154 cases were assessed using Metasin and GeneSearch. 37 cases (24%) containing 64 positive nodes were either positive for both CK19 and MGB or for one or the other (Tables 3 and 5). 111 cases (72%) containing 280 nodes were negative for both markers. Six cases (3.89%) were discordant, *i.e.*, the result of both assays did not match (Table 3). Four of those cases were Metasin-positive and GeneSearch-negative and two cases were Metasin-negative and GeneSearch-positive. Histological analysis of deeper levels revealed micrometastases in 3/4 cases, reinforcing the validity of the Metasin result (see Tables 3 and 5). Consequently, Metasin has a sensitivity of 95% (37/39, 95% CI 83%–99%) and a specificity of 97% with a discordance rate of 3.89%. Metasin has a PPV 90% and a NPV of 98%.

Figure 1 illustrates the distribution of Cq values obtained from the GeneSearch assay compared to Metasin. The individual Cq values for the predictive markers CK19 and MGB are presented in Figure 1A–C is a scatterplot of the distribution of Cq values for CK19 and MGB data obtained by Metasin, colour-coded and categorised as per macro-or micrometastasis by GeneSearch and histology. The scatterplot clearly illustrates the validity of the Metasin positive/negative and macro/micro Cq cut offs. Further description and discussion of the discordant nodes are available in the Table S1 and discussion sections, respectively.

### 2.6. Statistical Analysis: Comparison of Cq Values of Metasin with GeneSearch

In the positive node group, there was significant correlation between GeneSearch and Metasin for PBGD (rho = 0.499, *p <* 0.0001), CK19 (rho = 0.248, *p =* 0.041) and MGB (rho = 0.653, *p <* 0.0001). In the negative node group, there was significant correlation between GeneSearch and Metasin for PBGD (rho = 0.271, *p <* 0.0001) and MGB (rho = 0.471, *p <* 0.0001) but no significant correlation for CK19 (rho = −0.035, *p =* 0.567). Isolated tumour cells (ITCs) are considered negative and have a Cq value above the cut-off. The inclusion of this data impacts on the overall correlation for the negative node group and is most evident for CK19.

### 2.7. Independent Verification of Positive/Negative Macro/Micro Cut-Offs for Metasin

**Figure 2:**
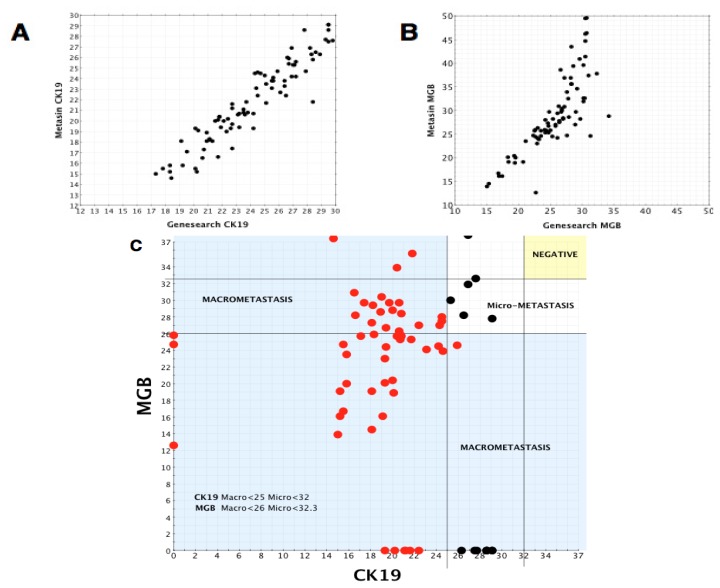
Comparison of Cq Values for GeneSearch *vs.* Metasin for JBI Samples. The scatter-plots illustrate Cq values for sentinel lymph node RNA samples examined by the Metasin assay. These results were compared to data obtained using GeneSearch A. (**A**) CK19 Cq values obtained for GeneSearch A plotted against the values for Metasin; (**B)** MGB Cq values obtained for GeneSearch A plotted against the values for Metasin; (**C**) The illustration shows the Cq values for Metasin determined CK19 (*x*-axis) *versus* MGB (*y*-axis). The coloured data points represent the differing combinations of the outcome for these nodes determined by Metasin. The Cq value cut-offs (indicated by solid lines) determined empirically for Ck19: Macros were less than 25 and micros more than 25 and less than 32: MGB Macros less than 26 and micros more than 26 and less than 32.3. The yellow shaded area (top right) represents nodes determined to be negative and the clear area represents micros as determined by Metasin. Nodes determined by GeneSearch as macros are illustrated as red data points and micros as black data points respectively.

RNA obtained from the JBI provided the opportunity to further refine the methodology and test the validity of the assay and cut offs. RNA samples from 193 anonymised (blinded to results) nodes were obtained. RNA had previously been prepared from these nodes as per GeneSearch protocol and assessed using the GeneSearch assay. Results were locally collated after re-analysis of these RNA samples on the Metasin assay. Table 6 and Figure 2A–C summarise the outcome for these samples. Of the 56 (29%) nodes categorised as macrometastases by GeneSearch, all were also correctly identified by Metasin. However, Metasin returned a negative result for 15 of the 33 nodes classified as micrometastases by Genesearch. These were mainly at the cusp of the micrometastasis/negative interface and generated Cq values 0.1 to 1.1 Cq from the cut-off boundary with the exception of one node, where the ΔCq was 2.1 between GeneSearch and Metasin (results not shown). All 104 (53.9%) nodes negative by Genesearch remained negative. The scatterplot illustrates CK19 and MGB Cq values obtained for GeneSearch compared with Metasin (Figure 2A,B). There is a significant level of correlation for CK19. For MGB, RNA samples tended not to show a linear relationship beyond a Cq value of 35.

The scatterplot in Figure 2C shows the distribution of the Cq values for CK19 and MGB data obtained by Metasin categorised as macro- or micrometastasis by previous GeneSearch analysis. The scatterplot clearly illustrates the validity of the Metasin positive/negative and macro/micro Cq cut offs.

### 2.8. Statistical Analysis of JBI Samples

JBI samples were analysed using the same statistical methods, comparing GeneSearch and Metasin Cq values for positive and negative node groups for CK19, MGB and PBGD.

Using the Spearman’s correlation test, there was significant correlation within the positive group between GeneSearch and Metasin for CK19 (rho = 0.842, *p <* 0.0001) and MGB (rho = 0.454, *p <* 0.0001) but no significant correlation for PBGD (rho = 0.177, *p =* 0.101). In the negative group, there was significant correlation for PBGD (rho = 0.383, *p <* 0.0001) and MGB (rho = 0.804, *p <* 0.0001), but there was no significant correlation for CK19 (rho = 0, *p =* 0.997).

### 2.9. CK19 and MGB IHC

All needle cores of the primary tumours analysed in the retrospective cases at Harlow (*n =* 154) were stained for CK19 and MGB and evaluated for staining characteristics. These cores were scored positive or negative. Consensus was universal except in 3 cases where there was arbitration by a third pathologist. Two cases failed to show tumours in the material submitted for immunostaining. Of the remaining cases (*n =* 152), 151 showed CK19 immunoreactivity. Conversely, only 86 of 152 cores (57%) showed MGB immunoreactivity. In two cases where the core biopsies showed intense immuno-reactivity for CK19, RNA from the matched positive lymph node failed to amplify for CK19 but Cq values for MGB were in the range characteristic for micrometastases.

#### Limits of the Detection of Metasin: Copy Number and Cell Count Number Determination

**Figure 3:**
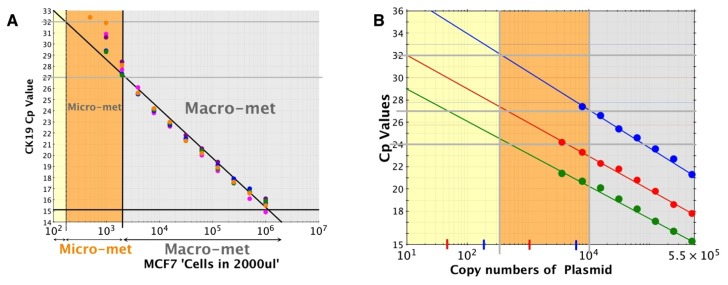
Metasin CK19 Cq values Cell Counts and copy numbers of plasmid. (**A**) The scatter plot illustrates the spread of Cq values in relation to the number MCF7 Cells harvested and examined by the Metasin assay. The data points correspond to cells harvested at 1 × 10^6^ cells. Doubling dilutions of RNA were prepared prior to Metasin. The *x*-axis shows the number of cells corrected for RNA dilutions corrected for number of cells in the diluted RNA samples. The vertical dotted line at 200 cells corresponds to presumed isolated tumour cell burden (ITCs) and the solid black line demarcates the boundary between Micro-metastasis and macro-metastasis tumour burden in the assay; (**B**) cDNA fragments cloned into plasmids prepared by TIBMOL BIOL were diluted at 10 fold dilutions and analysed by Metasin A. Cp values obtained (*y*-axis) are presented against dilutions. PBGD plasmid dilutions are green, CK19 red and MGB Blue.

The experiments were carried out at five different time points and doubling dilutions of RNA were used in the Metasin assay. Figure 3A illustrates a consistent linear relationship of “cell counts” to Cq value up to a Cq value of 27. Thereafter, the linear relationship between Cq value and cell count drift. Furthermore, up to this point the spread of Cq values for each of the 5 data points per dilution is of the order of 1 Cq value. Thereafter, the 5 repeated data points show a spread of 1.5 Cq points to 2.9 Cq points, reinforcing the lack of linearity at further dilutions of the cell line RNA. At 500-cell equivalence only one of five PCR reactions yields any signal. If this were extrapolated to the clinical scenario, then this would correspond to a detection capability of at least 1000 cells. Attempts to reproduce the doubling dilutions of MCF7 cells in normal lymph node homogenates failed to yield any meaningful results.

Cloned plasmids were separately diluted in 10 fold dilutions and evaluated on the Metasin assay. The copy numbers were plotted against Cq values corresponding to the dilution (Figure 3B).

## 3. Discussion

We describe the initial evaluation of Metasin in parallel with GeneSearch and compared with the benchmark histological assessment. The Metasin assay has many similarities to the withdrawn GeneSearch test, with a similar RNA preparation protocol and choice of predictive markers. It is fast, inexpensive, but can use any qPCR platform, unlike GeneSearch testing, which is software-limited to the Cepheid Smartcycler. Furthermore, in contrast to the commercial assay, we are reporting the primer and probe sequences, so allowing interested laboratories to reproduce and utilise this new assay. The assay’s high sensitivity, specificity and most importantly, capacity to deliver a result in less than 32 min makes it fit for the purpose of application in the intra-operative setting. Furthermore, since results appear in real-time, positive results can be reported as soon as the characteristic amplification plots appear.

Metasin makes use of the same strategy as the GeneSearch assay by including two markers for the prediction of metastatic disease, CK19 and MGB and reporting a positive result if both or either of the two markers generate amplification products. Published data [16,17] suggest that 98% to 99% of breast tumours are CK19 positive and extrapolate that the lymph node metastasis from these cases are also likely to be CK19 positive. However, no published data exist that correlate the immunohistochemistry data in the core biopsy or lymph node with the expression of CK19 at the RNA level. Hence it is interesting that during the development of the assay we scored 10% of cases with lymph node metastasis as CK19 negative at the RNA level. Fortunately, these cases were detected by both molecular tests since they were all positive for MGB. Two of these cases showed intense CK19 positivity by immunostaining of the core biopsies. These observations challenge the supposition that it is important to assess CK19 immunostaining of core biopsies for sentinel lymph node analysis [16,17]. Clearly, the use of molecular tests targeting CK19 only carries the clinical risk of missing nodal metastasis.

Assays targeting the CK19 gene can result in false positive results due to inadvertent amplification of CK19 pseudogenes, genomic DNA sequences similar to CK19 but not specifying translation products [18]. Traces of DNA that remain in RNA preparations can serve as templates for amplification with insufficiently validated primers, which are then detected with probes that are not sufficiently specific. Our approach essentially eliminates the risk of pseudogene amplification at two levels: (i) CK19 primer alignment shows several mismatches with both the forward and reverse primer binding sequences, making successful amplification of pseudogenes at the optimised annealing temperatures highly unlikely (see supplementary Figure provided); (ii) the detection probe also has a number of mismatches with pseudogene target sequences. Hence, even if a pseudogene were to be inadvertently amplified, the misamplified PCR amplicons would not be reported at the fluorescence detection temperature.

This was confirmed empirically in three ways:

1. amplification products run on agarose gels showed only a single band of the correct size (128 bp).
2. amplification of 100 DNA samples with the Metasin assay did not result in any detectable fluorescence signals and gel electrophoresis of amplified material yielded a series of smears.
3. We draw on our observations relating to the low discordant rates of the Metasin assay having analysed over 1700 cases maintaining an overall discordant rate under 4 percent.

Benchmarking the Metasin against the GeneSearch assay clearly demonstrates equivalence of results in most cases. Discrepancies of micrometastasis detection are probably accounted for by variable RNA degradation of the samples obtained from the JBI, which had been stored at −80 °C for more than two years and were used to validate the Metasin assay independently and blinded against the GeneSearch test. Our data reveal a discordance level of 3.98% between Metasin and histology, with a similar level (4.5%) of discordance observed for GeneSearch compared to histology. This is lower than previously reported discordance rates of intraoperative testing using qRT-PCR of up to 7% [5,9–11]. We have recently completed the validation of this assay in a much larger cohort of cases and demonstrate a discordance rate comparable to that reported here of <4% and the assay is now CE marked and available for general use (manuscript in preparation).

Although in diagnostic terms histopathology continues to be the gold standard, it has its limitations. A routine histopathological analysis usually analyses only a 3 μM slice from a wedge of tissue 2000 μM thick; the rest remains unexamined in the paraffin block. Consequently, an examination of sentinel nodes will detect metastases only if tumour is present in that particular 3 μM section. Furthermore, some histological subtypes (lobular carcinoma) are refractory to detection by the naked eye, leaving diagnosis dependent on detection by immuno-staining for cytokeratin [19]; hence guidelines for breast-cancer-reporting now mandate the use of immunohistochemistry [20].

To be absolutely certain of the absence of tumour in a lymph node, over 600 3 μM H & E stained sections would need to be carried out through a typical 2 mm lymph node slice [21]. This is clearly impractical, hence the compromise of examining the lymph nodes at three levels (three level, best practice) instead of examining one slide (single level analysis). Even then, this provides only a “snap-shot” view and most of the original 2 mm thick tissue will remain unexamined in the histology block, making the “gold standard” histological assessment rather inadequate.

Validation studies need to be bench-marked against current practice, which means single level histopathology of sentinel lymph nodes. Hence, alternative slices of serially sectioned lymph nodes were submitted for histology and for molecular analysis. Although examination of lymph nodes at three levels is adequate for most instances, it is not where “disease” is focal. Hence, given the focal nature of metastatic disease in lymph nodes, the separation of the segments makes some discordance between histology and molecular analysis inevitable. For example, one of the cases illustrated here shows a 2.1 mm macrometastasis that was missed by both Metasin and by GeneSearch. Examination of multiple levels revealed this to be focal disease with a macro-metastasis present in the horizontal plane, which cuts out after the examination of 2 levels (300 μM) and is not present in the third level.

Unlike PCR-based assays for sentinel node assessment, the OSNA assay available for intraoperative testing of sentinel nodes advocates the use of the whole node [22]. Here, we have demonstrated that the molecular approach can theoretically detect the presence of 500 to 1000 cells in lymph node homogenate without being subject to visual interpretive bias. Hence there is a rational argument for using the whole node for molecular analysis. However, this leaves no reserve material available for review and so requires any molecular assay to be utterly reliable. Metasin failed to detect three macro-metastasis (see Table provided in supplementary data), two of which also tested negative by the Genesearch test, suggesting that this discordance was due to allocation bias (see Table provided in supplementary data) and that the assays would have detected the tumour had the whole node been analysed. One was detected by the Genesearch assay, with the reason for this discrepancy unclear. Assay failure occurs in around 1.5% of cases (manuscript in preparation) and there is a rare theoretical risk of false positives due to the presence of epithelial inclusions [23,24] or the expression of CK19 in some high-grade lymphomas [25,26]. Clearly, these limitations must be highlighted in guidance notes to clinicians.

The sensitivity of the assay was determined by using doubling dilutions of cell line RNA or plasmid DNA, since the control MCF7 cell line expresses only CK19 and PBGD, but not MGB. The assay is capable of detecting as few as 200 cell equivalents of RNA, although, this is not consistently reproduced. The significance of these observations is that ITCs are estimated to be of the order of 200 to 500 cells since counts more than this most likely represent micro-metastasis [5,27,28].

## 4. Experimental Section

### 4.1. Ethical Approval/PAH Samples

This study was ethically approved by the Essex 2 Research Ethics Committee (Ethics Approval Reference: 07/H0302/129 & REC 10/H301/24). All patients undergoing sentinel lymph node biopsy for breast cancer during March 2008 to July 2010 at PAH were consented for their sentinel lymph node tissue to be tested using the GeneSearch and Metasin tests. The inclusion criteria for participants included those undergoing a surgical excision of a primary operable breast cancer and sentinel lymph node sampling. No exclusion criteria were named. The patients were operated on by surgeons after the implementation of the “NEW START” program [4]. The results of primary data relating to the GeneSearch and the histology were known to the operators before validating the assay.

### 4.2. Histopathological Processing and Assessment of Sentinel Lymph Nodes

The sentinel lymph nodes were identified by a combined technique of radioactive (TcM99m) labelled nanocolloid and 2 mL of diluted Patent V blue dye [29]. Excess fat was removed from the lymph nodes and they were sliced into 2 mm sections with alternate slices allocated to Genesearch/Metasin and histology assessment. Slices for histology were processed individually according to local histology protocol and involved cutting five levels of 100 μM each together with three unstained serial sections per level. The slides were examined by two experienced Consultant Histopathologists and discordant node analysis included examination of a further five levels with immunostaining for MNF116 [30].

### 4.3. Node Processing for GeneSearch/Metasin

RNA extractions of the sentinel lymph node slices were carried out using a locally modified protocol of Qiagen’s RNeasy mini RNA extraction kit. The lymph node weight used for the RNA extraction step is the most critical parameter and it was essential to use the exact volume of homogenisation buffer [31]. The weight of each lymph node was determined very precisely, with the same amount used for every extraction. Tissue was homogenised in 400 μL Buffer RLT, an equal amount of 70% ethanol was added to the homogenised tissue, the tubes were vortexed briefly and the homogenate-ethanol mixture was added to RNeasy mini columns attached to a vacuum. Following several washes in proprietary Qiagen buffers, the RNA was eluted in 50 μL of RNAse-free water by spinning the columns in a microfuge at 10 × G for 1 mine.

### 4.4. Ethical Approval/JBI RNA

NREC project REC 10/H301/24 covered ethical approval for RNA obtained from the JBI. Stored frozen (−80 °C) RNA from 193 nodes were obtained retrospectively. These had been analysed previously using the GeneSearch assay, which recorded 104 nodes as negative and 89 as positive. The demographic details, histological outcome and axillary clearance data for this grouping were incomplete. The operators were blinded to the results of the original GeneSearch assay, which were provided after conclusion of the analysis.

### 4.5. Quantitation and Identification of Template (RNA and DNA)

A Nanodrop microvolume spectrophotometer (Thermo Scientific, Wilmington, DE, USA) was used to quantify all RNA samples. Purity was assessed using 260/280 ratios and, in addition, the RNA quality of a selection of random samples was assessed using a Bioanalyzer 2100 (Agilent, Stockport, UK).

### 4.6. Primer and Probe Sequences

Target sequences for PBGD (NM_000190), CK19 (NM_002276) and MGB (NM_002411) were obtained from the National Centre for Biotechnology Information (NCBI) GenBank sequence database (http://www.ncbi.nlm.nih.gov/genbank/). qPCR amplification primers and hydrolysis probes were designed by a specialist oligonucleotide supply company (TIB Molbiol, Berlin, Germany) (Table 7). All primers were located in different exons, thus minimising the risk of genomic DNA amplification and great care was taken to ensure that pseudogenes [18] are not amplified (supplementary data). Primer annealing temperatures ranged from 56.2 to 57.9 °C.

### 4.7. RT-PCR Assay

All PCR assays were performed on the SmartCycler 2.0 (Cepheid, Sunnyvale, CA, USA) with the aim of generating quantification cycles (Cqs) for Metasin that are close to those of the Genesearch test. Hence, optimisation matrices of primer (1.0–2.5 μM) and probe (0.05–0.1 μM) concentrations, together with optimised RNA concentrations were used to identify the optimal combination of oligonucleotide and RNA concentrations. The final protocol was a one-tube, two-step protocol that makes use of the dual RNA- and DNA-dependent polymerase activities of Tth DNA polymerase. Reactions used 4 μL RNA in a total reaction volume of 25 μL with final primer and probe concentrations of 2.5 μM and 1.0 μM, respectively and were run in parallel by a Biomedical Scientist (BMS) (PSG) and a research fellow (SAR). The RT step of the two-step protocol was carried out for 4 min at 60 °C, followed by 40 cycles of 95 °C 10 s, target-specific annealing temperature for 10 s and a polymerisation step at 72 °C for 10 s. The details of the reporting of the RT-qPCR experiments described here are compliant with the MIQE guidelines [32].

### 4.8. Positive and Negative Controls

Every run contained at least one positive control and one negative control. The positive control consisted of a sample of RNA that had previously tested positive for all three mRNA targets; the negative control was an RNA sample that had previously tested negative for CK19 and MGB, but positive for PBGD.

### 4.9. PCR Efficiency and Copy Number Calculations

The MCF7 (ATCC) cell line was grown in Eagle’s MEM culture medium supplemented by 10% FBS in 5% CO_2_[33]. On reaching 70–80 percent confluence, cells were trypsinised, harvested and counted. One million cells were used for RNA extractions using the modified RNA extraction protocol described above. RNA was two-fold serially diluted, 4 μL of each serially diluted RNA was used with the Metasin assay and resulting Cqs were plotted against the log of RNA concentration. This was repeated five times and results are shown in Figure 3A.

To correlate Cq with copy number, linearised plasmids specifying the amplicons for CK19, MGB or PBGD were prepared by TiBMOL BIOL at 4 × 10^9^ copies/μL. They were ten-fold serially diluted, amplified using the Metasin assay and resulting Cq values were plotted against the log of the copy numbers (Figure 3B).

### 4.10. Cut-Off Cq Value for CK19 and MGB for Positive and Negative Nodes

A scatter plot was prepared plotting Metasin Cq values for CK19 *vs.* MGB of all nodes. Each point on the scatter plot was also correlated with histology. Vertical and horizontal lines were drawn selecting the best Cq value for both markers which incorporated all the true positive nodes without creating any false-positive node results. Once these cut-offs were established, their sensitivities along with the confidence intervals and specificities were calculated as well as their negative and positive predictive values. The cut off Cq value for each marker was chosen so as to minimise the number of false positive results.

The same method was used to determine the optimum cut-offs for micrometastases and macrometastases.

### 4.11. Statistical Analysis

The data were shown to be non-parametric, with the Kolmogorov-Smirnoff test of distribution indicating a uniform but non-Gaussian distribution (*Z* value = 2.187, *p* value < 0.0001). Cq values were separated according to positive and negative nodes and comparison of Genesearch and Metasin for PBGD, CK19 and MGB was performed using Spearman correlation tests. The null hypothesis was defined as there not being any significant difference between Genesearch and Metasin Cq values for each of the three markers.

### 4.12. CK19 and MGB IHC on Breast Cores

All needle cores of the cases analysed in the retrospective cases at Harlow were stained for CK19 and MGB evaluated for staining characteristics. These cores were scored positive or negative by two experienced Consultant Histopathologists (EA & MLS). The Consultant Histopathologist reviewing the slides were “blind” to the outcome of the Metasin and GeneSearch data.

## 5. Conclusions

The Metasin assay is fast fit for purpose with a discordance rate below 4% ready for implementation in the intraoperative setting for the assessment of sentinel nodes.

## Supplementary Information

Figure S1. PCR product identification. PCR products from individual PCR reactions were size fractionated using a 4 percent gels (Nusieve) and prepared in TBE buffer. Ten micro-litres of PCR product was run in each lane and gel stained with ethidium bromide and the gel was photographed under UV light. The upper panel (**A**) illustrate the fluorescence of the PCR products from PCR amplification of PBGD (Lanes 1–3), CK19 (4–6) and MGB (lanes 7 & 8). Lanes 9 to 13 are PCR reactions from known positive cases. The molecular weight markers (MW = phix Hae III digest markers) are indicated to the left of the panel A and also within the gel (lane 7). The size markers correspond to CK19 with 128 base pairs (bp), PBGD 92 bp and MGB 69 bp. The (**B**) illustrates the PCR bands corresponding to each of the relevant genes (labelled (PBGD, CK19 & MGB). A schematic is shown to the right (last panel) illustrating the band sizes corresponding each of the 3 genes with their expected band sizes to the right of the schematic. The penultimate panel illustrates the 10 μL of the Metasin BLN PCR product.

Figure S2. CK19 Pseudogene Alignments. CK19 Pseudogene alignments [18] (**A**) Sequence alignment of members of the CK19 gene family. CK19. Primer alignment (**B** & **D**) shows several mismatches with both the forward and reverse primer binding sequences. The detection probe sequence (**C**) also has a number of mismatches with pseudogene target sequences.

**Table S1.** Summary of discordant case analysis. Metasin *vs.* GeneSearch and Metasin *vs.* Histology—Discordance analysis.

Case No	Metasin	GeneSearch BLNA	Histopathology		Node-based Concordance or Discordance	Case-based- Concordance/discordance	Notes
			Initial Analysis	Deeper Levels			
^1^ 16837	Negative	**Macro-metastasis**	**Macro-metastasis**	NA	**Macro-metastasis Discordant**	**Macro-metastasis Discordant**	^1^ 2008 case-earliest batch of cases (? RNA degradation) Macro detected in one of 2 slices given for histology in 2 levels only. Not present in third slice or in deeper level
^2^ 7587B	Negative	Negative	**Macro-metastasis**	NA	**Macro-metastasis Discordant**	**Concordant**	^2^ Another lymph node from this case showed both histological and molecular evidence of metastatic disease-hence not a true discordant case. ^3^ Macro-metastasis 3 mm tumour in 1 slice
^3^ 12395	Negative	Negative	^3^ **Macro-metastasis 3 mm in 1 slice**	NA	**Macro-metastasis Discordant**	**Macro-metastasis Discordant**	^3^ Macro-metastasis 3 mm tumour in 1 slice
^4^ 12082	Positive Micromet	Negative	Negative	Negative	**Micro-metastasis Discordant**	**Micro-metastasis Discordant**	^4^ Micro met MGB 31.5, Metasin Cq for CK19 is 29.9 (Veridex cut off is 30)
^6^ 9041B	^6^ Positive Micromet	^6^ Positive Micromet	Negative	Negative	**Micro-metastasis Discordant**	**Micro-metastasis Discordant**	^6^ 9041B Cp MGB is 30.4, Metasin 29.8. Positive independent of Metasin on GeneSearch BLN Assay
^7^ 8489	Positive Micromet	Positive Micromet	Negative	^7^ ITC	**Micro-metastasis Discordant**	^7^ **ITC’s present in axillary clearance**	^7^ ITCs seen in deeper levels and in axillary clearance specimen. Positive independent of Metasin on GeneSearch BLN Assay
^8^ 10931B	**Positive Macro**	Positive Micromet	Negative	Negative	**Micro/Macro metastasis Discordant**	**Micro/Macro metastasis Discordant**	^8^ Positive independent of Metasin on GeneSearch BLN Assay

## Figures and Tables

**Table 1:** Patient demographics and clinical information.

	Nodal status	All patients	Total	Type of tumour	Nodal status		Total
Patients Enrolled age, years		156		IDC	positive SN	26	103
					negative SN	74	

mean age at diagnosis	positive SN	60.2	62.2		discordant SN	3	
	negative SN	63.2		ILC	positive SN	6	16
	discordant SN	60.2			negative SN	9	

median age at diagnosis	positive SN	59			discordant SN	1	
	negative SN	63	63	Others	positive SN	6	32
	discordant SN	62			negative SN	24	

age range	positive SN	39–92			discordant SN	2	
	negative SN	34–87	34–92	DCIS	positive SN	0	11
	discordant SN	43–68			negative SN	10	

Surgery Performed					discordant SN	1	

Mastectomy	positive SN	18			positive SN	0	
	negative SN	18	42	LCIS	negative SN	1	1
	discordant SN	6			discordant SN	0	

Wide local excision	positive SN	18	100	Hormone receptor status			
	discordant SN	0			negative SN	17	

Tumour Size (TNM) (*15*)					discordant SN	0	
pT1 (<2 cm)	positive SN	18			positive SN	7	
	negative SN	61	79	Mean ER score	negative SN	6	7
	discordant SN	0			discordant SN	7	

pT2 (>2 cm, <5 cm)	positive SN	15			positive SN	27	
	negative SN	28	46	PR positive	negative SN	71	103
	discordant SN	3			discordant SN	5	

pT3 (>5 cm)	positive SN	2			positive SN	10	
	negative SN	2	4	PR negative	negative SN	34	45
	discordant SN	0			discordant SN	1	

Tumour Grade				Mean PR score	positive SN	6	5

Grade 1	positive SN	5			negative SN	5	
	negative SN	15	20	Her2 positive	discordant SN	5	
	discordant SN	0			positive SN	4	13

Grade 2	positive SN	20	78		negative SN	8	
	negative SN	55		Her2 negative	discordant SN	1	
	discordant SN	3			positive SN	33	127

Grade 3	positive SN	12	29		negative SN	90	
	negative SN	16		Triple negative	discordant SN	4	
	discordant SN	1			positive SN	1	14

Ungradeable	positive SN	0	10		negative SN	13	
	negative SN	9			discordant SN	0	
	discordant SN	1					

**Table 2:** Discordant analysis and case-based outcome after deeper levels.

	Concordant cases		Discordant cases		Total	Discordant cases
	H+ M+	H− M−	H+ M−	H− M+		

Initial analysis	33 (21.4%)	111 (7.2%)	2 (1.3%)	8 (5.2%)	154	10 (6.49%)
Deeper levels	37 (24%)	111 (77%)	2 (1.3%)	4 (2.6%)	154	6 (3.89%)

H = Histology, M = Metasin: + = positive and − = negative.

**Table 3:** Results of Metasin for cases and nodes compared with GeneSearch A (**left**) and Histology (**right**).

Metasin *vs*. GeneSearch A		Metasin *vs*. Histology	
Node Based-Analysis		Node Based-Analysis	

Positive nodes	64 (18.2%)	Positive nodes	61 (17.4)
Negative nodes	280 (80%)	Negative nodes	280 (80%)
Discordant nodes	6 (1.7%)	Discordant nodes	9 (2.5%)
Total	350	Total	350

Case Based-Analysis		Case Based-Analysis	

Positive patients	37 (24%)	Positive patients	37 (24%)
Negative patients	111 (72%)	Negative patients	111 72%
Discordant patients	6 (3.89%)	Discordant patients	6 (3.89%)
Total	154	Total	154

**Table 4:** Comparison of tumour volumes in concordant positive nodes between Metasin and histology (*n =* 61).

	Metasin		Total
Histology	Macro	Micro	
Macro	50 (81.9%)	2 (3.2%)	52 (85.2)
Micro	1 (1.6%)	8 (13%)	9 (14.7)
Total	51 (84%)	10 (16.4%)	61 (100%)

**Table 5:** Discordant results between Metasin and GeneSearch A.

Node based discordance					
Concordant		Discordant		Total	Discordant nodes

1 G+ 2 M+	G− M−	G+ M−	G− M+		
64 (18.2%)	280 (80%)	2 (0.6%)	4 (1.14%)	350	6 (1.17%)

Case based discordance					

Concordant		Discordant		Total	Discordant cases

G+ M+	G− M−	G+ M−	G− M+		
37 (24%)	111 (72%)	2 (1.2%)	4 (2.6%)	154	6 (3.89%)

1 G = GeneSearch A,

2 M = Metasin: + = positive and − = negative.

**Table 6:** Summary of Metasin analysis of Jules Bordet Institute (JBI) GeneSearch assayed lymph nodes.

	Genesearch analysed nodes from JBI 1			Total
	Macro-Metastasis 1	Micro-metastasis	Negative Nodes	
Metasin Positive Nodes 2	56 (29%)	18 (9.3%)	–	74 (38.3%)
Metasin Negative Nodes	–	15 (7.7%)	104 (54%)	119 (61.7%)
Total	56 (29%)	33 (17.1%)	104 (53.9%)	193 (100%)

1 Genesearch based macro/micro cut offs were defined as CK19 < 25 for macro-metastasis and less than 30 for micro-metastasis. MGB < 26 for macro-metastasis and less than 31 for micro-metastasis.

2 Metasin cut offs were defined as CK19 < 25 for macro-metastasis and less than 32 for micro-metastasis. MGB < 26 for macro-metastasis and less than 32.3 for micro-metastasis.

**Table 7:** Primer and probe sequences used in the Metasin assay.

Marker	Hydrolysis probe	Amplicon length	Forward primer 5′ to 3′	Reverse primer 5′ to 3′
PBGD 1	ctcctgaactccagatgcggga	92	tgtggtgggaaccagctc	tgttgaggtttccccgaat
CK19 2	cagccagacgggcattgtcg	128	cagccactactacacgaccatc	caaacttggttcggaagtcatc
MGB 3	ctctggctgccccttattggag	69	ctcccagcactgctacgc	ggattgattgtcttggaaatcaca

Dye label:

1 cyan 500;

2 LC610;

3 LC670.
