# Supplementary figures and images for: Metasin—An Intra-Operative RT-qPCR Assay to Detect Metastatic Breast Cancer in Sentinel Lymph Nodes
Source: Int J Mol Sci. 2013 Jun 24;14(7):12931–52. doi: 10.3390/ijms140712931 (PMC3742166; doi:10.3390/ijms140712931)

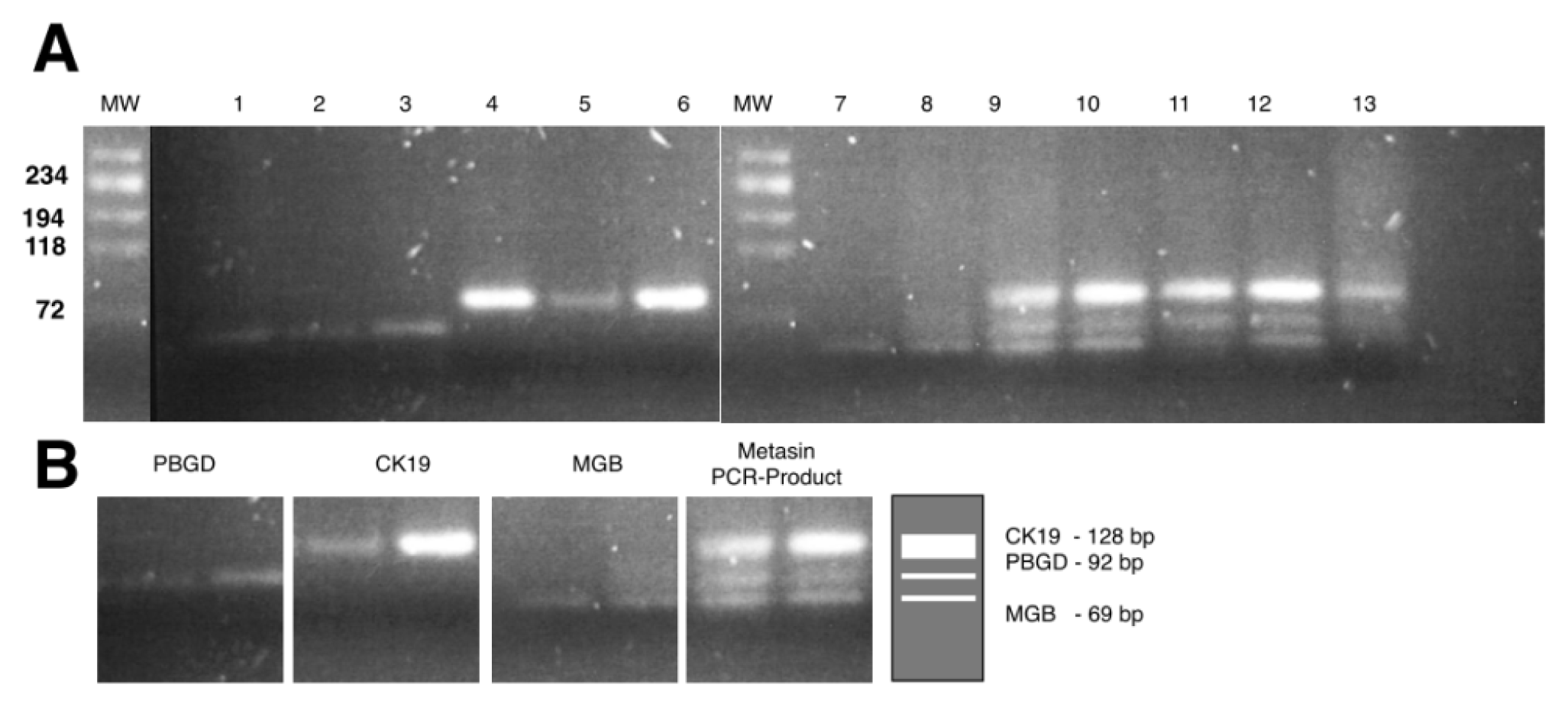

Supplement: Figure S1. — PCR product identification. PCR products from individual PCR reactions were size fractionated using a 4 percent gels (Nusieve) and prepared in TBE buffer. Ten micro-litres of PCR product was run in each lane and gel stained with ethidium bromide and the gel was photographed under UV light. The upper panel (A) illustrate the fluorescence of the PCR products from PCR amplification of PBGD (Lanes 1–3), CK19 (4–6) and MGB (lanes 7 & 8). Lanes 9 to 13 are PCR reactions from known positive cases. The molecular weight markers (MW = phix Hae III digest markers) are indicated to the left of the panel A and also within the gel (lane 7). The size markers correspond to CK19 with 128 base pairs (bp), PBGD 92 bp and MGB 69 bp. The (B) illustrates the PCR bands corresponding to each of the relevant genes (labelled (PBGD, CK19 & MGB). A schematic is shown to the right (last panel) illustrating the band sizes corresponding each of the 3 genes with their expected band sizes to the right of the schematic. The penultimate panel illustrates the 10 μL of the Metasin BLN PCR product. [file ijms-14-12931s1.tif]

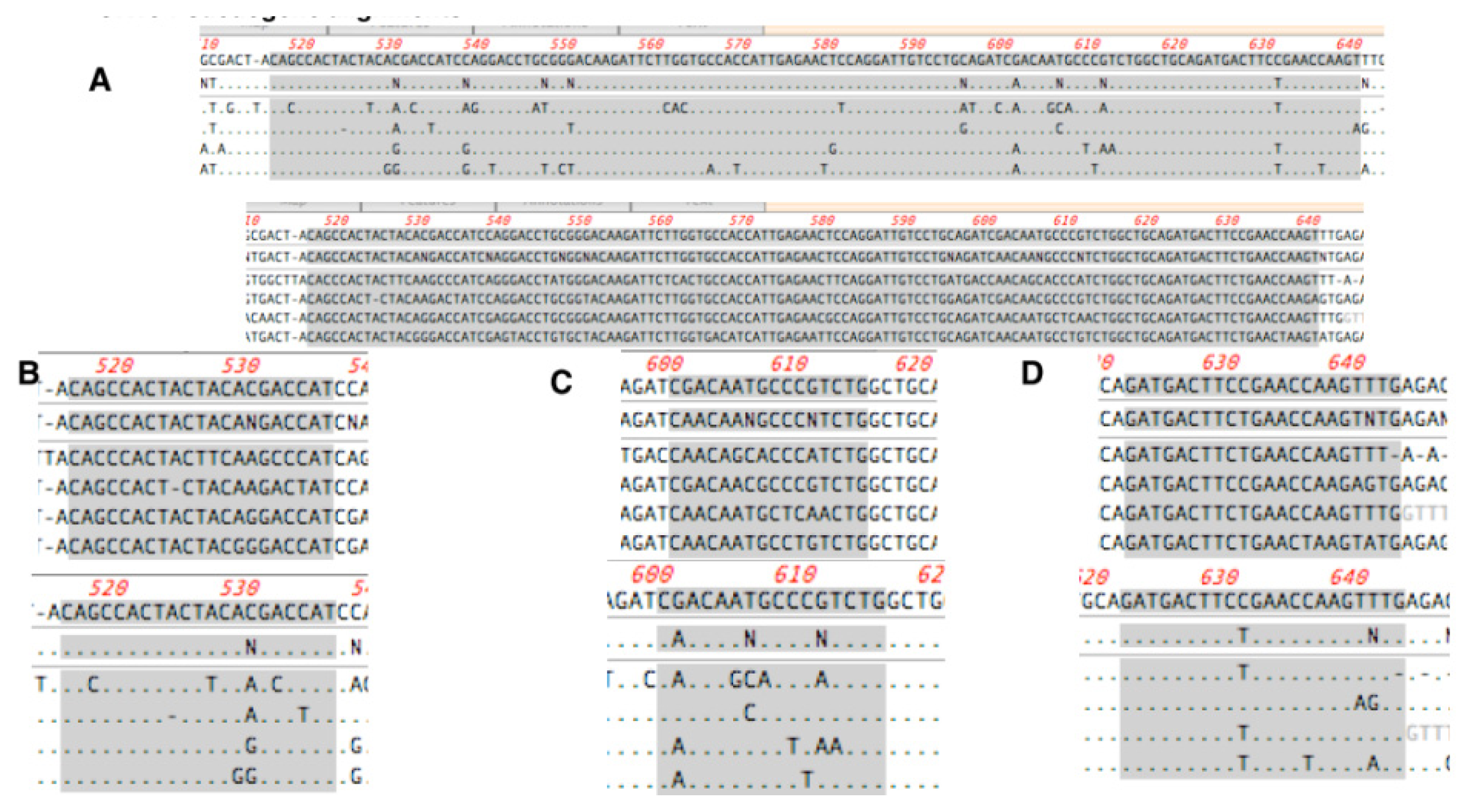

Supplement: Figure S2. — CK19 Pseudogene Alignments. CK19 Pseudogene alignments [18] (A) Sequence alignment of members of the CK19 gene family. CK19. Primer alignment (B & D) shows several mismatches with both the forward and reverse primer binding sequences. The detection probe sequence (C) also has a number of mismatches with pseudogene target sequences. [file ijms-14-12931s2.tif]
